# Factors associated with access to HIV care services in eastern Uganda: the Kumi home based HIV counseling and testing program experience

**DOI:** 10.1186/s12875-015-0379-6

**Published:** 2015-11-03

**Authors:** David Lubogo, John Bosco Ddamulira, Raymond Tweheyo, Henry Wamani

**Affiliations:** Department of Community Health and Behavioural Sciences, Makerere University, College of Health Sciences, School of Public Health, P.O.Box 7072, Kampala, Uganda; Department of Disease Control and Environmental Health, Makerere University, College of Health Sciences, School of Public Health, Kampala, Uganda; Department of Health Policy Planning and Management, Makerere University, College of Health Sciences, School of Public Health, Kampala, Uganda

**Keywords:** Access, HIV care services, Cotrimoxazole prophylaxis, Home based counseling and Testing

## Abstract

**Background:**

The HIV/AIDS health challenge continues to ravage many resource-constrained countries of the world. Approximately 75 % of all the global HIV/AIDS related deaths totaling 1.6 (1.4–1.9) million in 2012 occurred in sub-Saharan Africa, Uganda contributed 63,000 (52,000–81,000) to these deaths. Most of the morbidity and mortality associated with HIV/AIDS can be averted if individuals with HIV/AIDS have improved access to HIV care and treatment. The aim of this study therefore, was to explore the factors associated with access to HIV care services among HIV seropositive clients identified by a home based HIV counseling and testing program in Kumi district, eastern Uganda.

**Methods:**

In a cross sectional study conducted in February 2009, we explored predictor variables: socio-demographics, health facility and community factors related to access to HIV care and treatment. The main outcome measure was reported receipt of cotrimoxazole for prophylaxis.

**Results:**

The majority [81.1 % (284/350)] of respondents received cotrimoxazole prophylaxis (indicating access to HIV care). The main factors associated with access to HIV care include; age 25–34 years (AOR = 5.1, 95 % CI: 1.5–17.1), male sex (AOR = 2.3, 95 % CI: 1.2–4.4), urban residence (AOR = 2.5, CI: 1.1–5.9) and lack of family support (AOR = 0.5, CI: 0.2–0.9).

**Conclusions:**

There was relatively high access to HIV care and treatment services at health facilities for HIV positive clients referred from the Kumi home based HIV counseling and testing program. The factors associated with access to HIV care services include; age group, sex, residence and having a supportive family. Stakeholders involved in providing HIV care and treatment services in similar settings should therefore consider these socio-demographic variables as they formulate interventions to improve access to HIV care services.

**Electronic supplementary material:**

The online version of this article (doi:10.1186/s12875-015-0379-6) contains supplementary material, which is available to authorized users.

## Background

The HIV/AIDS health challenge continues to ravage many resource-constrained countries of the world. The morbidity and mortality due to HIV/AIDS is highest in sub Saharan Africa. As of 2013, 24.7 (23.5–26.1) million of the 35 million people living with HIV globally were in sub-Saharan Africa [1]. Approximately 75 % of all the global HIV/AIDS related deaths totaling 1.6 (1.4–1.9) million in 2012 occurred in sub-Saharan Africa and Uganda contributed 63,000 (52,000–81,000) to these deaths [2]. Notably, there was a decline in the number of AIDS related death in sub-Saharan Africa by 39 % between 2005 and 2013, a success that was directly attributed to the increase in the number of people on antiretroviral therapy (ART) [1].

Improving access to HIV treatment has the potential of saving lives [3–5]. However, programs offering HIV/AIDS counseling and testing require linkage and retention onto care and treatment of patients who test HIV positive for positive outcomes. In Uganda, designated health facilities/chronic HIV care clinics offer comprehensive HIV/AIDS care, treatment and support services to the community. The services offered include: HIV/AIDS counseling and testing (HCT), clinical, psychosocial, nutritional, legal, economic, family social and community support for clients [6, 7]. Cotrimoxazole prophylaxis is an essential component of care which reduces morbidity and mortality among HIV positive persons [8]. The Ugandan Ministry of Health treatment guidelines therefore recommends that all people living with HIV/AIDS take cotrimoxazole prophylaxis, irrespective of whether they are on ART or not [9]. Thus early diagnosis of HIV infection through HIV counseling and testing and linkage to HIV care facilities is essential in improving the prognosis and survival of HIV infected individuals [10].

Some studies conducted in Uganda [11] and other African countries [12–14] have reported a low linkage to HIV care of newly diagnosed HIV sero-positive individuals. While HCT has a significant impact on linkage to care [11], its uptake has largely remained limited [2]. This has hindered attempts to prevent new HIV infections and scale-up of HIV care and treatment for people living with HIV globally [15]. Factors at individual, system and community levels may influence care seeking in an individual who has tested HIV positive [16]. At individual level, women access HIV care services better than men [2, 17–19]. However, other researchers have suggested otherwise [20]. Some studies have noted distance to the facility as an important factor [21, 22]. The presence of a supportive partner/ spouse may positively influence access to care services [16, 23, 24]. On the other hand, disclosure of sero status to family members, including their main partner may present a barrier to seeking care [25]. Some studies have also noted the positive role played by a supportive community to enable HIV patients’ access HIV care services [23, 26–28].

This study was conducted in 2009 within a well-established district-wide HIV home based counseling and testing (HBCT) program, the Kumi HBCT program. The program used a home-based HCT delivery model [6, 29, 30], and was implemented by the Kumi district local government between 2006 and 2008. The United States of America’s Centers for Disease Control and prevention (CDC), Atlanta and the United States President’s Emergency Plan for AIDS Relief (PEPFAR) provided the financial support. The goal of the program was to offer HCT to the entire population resident in Kumi district, eastern Uganda and refer all those with HIV infection to health centres for ongoing psychosocial support, basic preventive, palliative care and treatment services.

Prior to 2006, only three health facilities in the district offered voluntary counseling and testing (VCT) services for HIV, later termed HCT. The demand for VCT services was high and yet the health system lacked adequate human resource and logistics like testing kits. Therefore, the Kumi HBCT built capacity of local volunteers/ health providers whom they equipped with logistics to support HCT in the district. Preliminary preparations involved training of counselors, laboratory technicians, team supervisors and community owned resource persons (CORPs) in HBCT for HIV [29]. CORPs are a specific type of community volunteers that were trained and utilized mainly during the scale-up of the national HIV program countrywide, during 2001 to 2010. Over 200 CORPs were trained on basic counseling and community mobilization in Kumi district. The CORPs tasks/roles included; registering households and preparing them for HBCT services. At times CORPs conducted follow up visits for clients who had disclosed their sero-status to the CORPs. During the visits, the CORPs sensitized the HIV positive clients about the need to seek care and treatment at health facilities. The health workers at the health facilities were trained on how to provide basic care services including the HIV basic care package (BCP) to HIV positive clients referred to them by the outreach teams. The HBCT program also encouraged the HIV positive individuals (peer educators) to form groups (post-test clubs and drama groups). These groups held drama shows during which HIV/AIDS related messages were disseminated to the community members. Key messages included, adherence to treatment, encouragement of disclosure, uptake of the Basic Care Package, avoidance of stigmatizing HIV positive individuals and the importance of seeking care at health facilities when sick. The drama performances were normally followed by HIV/AIDS educational talks delivered by a trained counselor to the community members. The talks were meant to supplement on the presentations by the peer educators.

The counselors and laboratory technicians were based at sub county level with a catchment population of 200,000 while CORPs were based at parish level with catchment population of 5,000. Community mobilization activities (weekly talk shows, quarterly film shows and the use of local leaders and CORPs to encourage HBCT activities uptake was undertaken prior to the team visits. The CORPs were responsible for guiding the HBCT field teams to the client homes. In a home, household members were offered education on basic facts about HIV/AIDS, STDs and benefits of HBCT and importance of disclosure. Eligible household members who consented to the service were given pre-test counseling either individually or as a couple. Couple counseling was provided to partners who were married or cohabiting and wished to be counseled together. Thereafter blood was drawn and tested following a nationally standardized rapid serial HIV testing algorithm. The clients were given their HIV test results during the post-test counseling session on the same day of the test. [6] While risk assessment was done separately, the couple was introduced to the test results and encouraged to share or disclose the results of the tests. At the time of HIV testing, blood was also taken off and saved for CD4 cell count testing at the CDC, Tororo. The results were then sent online to the Kumi HBCT Data Manager in approximately a week’s time. In case the laboratory at CDC, Tororo was not operational for whatever reasons, the samples would then be taken to CDC, Entebbe. Under the prevention of mother to child transmission (PMTCT) of HIV program, dry blood spot samples were taken off from children of HIV positive mothers and sent via post office to the Joint Clinical Research Centre (JCRC), Kampala for HIV DNA polymerase chain reaction (PCR) testing.

Following HIV testing, all HIV positive clients were referred to their nearest health facilities for chronic HIV care as they awaited the results of the CD4 cell count tests. The HBCT field teams were responsible for delivering the results of the CD4 cell count testing and the HIV DNA PCR testing to the client’s homes. They would explain the results to the clients and then ask the clients to go back to the health facilities with the results to obtain the appropriate care and treatment. Some of the services at the referral facilities included: cotrimoxazole prophylaxis, TB screening, ART assessment, and the provision of a basic care package (BCP) comprising of; a safe water vessel, insecticide treated mosquito net, condoms and information, education and communication (IEC) materials such as PMTCT leaflets for pregnant mothers.

Household and client information was collected using household census forms and client profile forms respectively. Each client’s data was entered and stored in a computer by data entrants at the head office. Each client record had a unique identifier and the records were kept under the overall supervision of the HBCT data manager. During the implementation of the project in the district, teams moved from door to door and covered one village before moving to the next village in a systematic manner.

The information from the project data manager indicated that about 64.6 % of all HIV positive clients referred from the Kumi HBCT program reached the facilities. Little was known about factors that influenced access to HIV care and treatment services in this rural community. Although some studies have been carried out in the area of access to HIV care services in Uganda, most of these studies were done in urban areas [10, 11], at health facilities [10, 11], explored the timing of initiation/entry into care for services such as ART [10, 31] and were qualitative by design [32]. There are few studies that have been carried out on access to HIV care at population level in a rural setting in Uganda and other resource limited countries. Therefore the aim of this study was to explore the factors associated with access to HIV care services by HIV positive clients in a rural setting in eastern Uganda.

## Methods

### Study setting

The study was conducted in January and February 2009 in Kumi district, eastern Uganda. The district has a population of 341,393 people, a population growth rate of 4.3 % and HIV estimated prevalence of 3.6 %. The district has 27 health facilities (3 hospitals, HCIV’s, HCIII’s and HCII). All the HC III’s, HC IV’s and hospitals in the district offer cotrimoxazole prophylaxis to HIV positive clients however ART services are offered by the HCIV’s and Hospitals. Other HIV care services offered in the district include: PMTCT, VCT, and STD/ HIV control. The AIDS information centre (AIC) and the Kumi AIDS support organization (KASO) also operate in the district.

### Study design and population

This was a cross-sectional study where quantitative data was collected using semi-structured questionnaires. The study population consisted of HIV positive clients aged ≥18 year’s old, resident in Kumi district and registered with the Kumi HBCT program between 2006 and 2008. Only individuals who gave informed consent and were willing to participate were included in the study.

### Study sample, data collection and analysis

We calculated the sample size using the formula for Kish Leslie (1965) [33]. The parameters used for sample size calculation were: 64.6 % estimated proportion of clients who received cotrimoxazole prophylaxis (2009), 5 % maximum marginal error at 95 % confidence interval. A sample size of 352 respondents was obtained. The outcome variable was reported receipt of cotrimoxazole Additional file 1.

We enumerated a sampling frame of 5044 HIV positive clients registered for the first time (non-repeat testers) with the Kumi HBCT program. A sampling interval of 15 was obtained by dividing 5044 by the sample size of 352. We selected the first client name in the sample randomly using computer generated random numbers from the first 14 names in the fixed list containing the 5044 client names arranged alphabetically and numbered from 1 to 5044. Thereafter, every 15th name on the list was selected. Systematic sampling was continued until all the 352 participants were obtained. This was the first level of selecting clients.

At the second level, the 352 names in the sample were then traced in the community. In case a sampled client was not found in their expected household, attempts were made to trace them using information from their neighbors’ and through telephone calls.

The CORPs were used to locate the client’s homes because they possessed local knowledge of the area and were believed to be more acceptable to the community members.

Their involvement in the study did not draw unnecessary attention to the study subjects, since the community members considered the CORPs to be conducting their routine work in the community. Upon identification, clients were invited to participate in the survey after obtaining written informed consent. Four trained bilingual research assistants administered a pre-tested, semi-structured questionnaire to the clients in the local language, Ateso. The interviews were conducted in a quiet environment at the client’s home and lasted approximately 45 min. No incentives were provided for clients to participate in the interviews. Clients who were extremely ill to respond to interviews were excluded at this level.

### Measurement of variables

The main outcome variable (access to HIV care services) was measured by the reported receipt of cotrimoxazole for prophylaxis from an ART/HIV clinic by clients registered at the ART/HIV clinics in a given facility. Cotrimoxazole is given to all HIV positive clients who have not progressed to the use of ART, and those on ART. [9] It was thus considered to be an appropriate measure of access to HIV care services and was assessed as a binary variable using closed questions: ‘did you receive cotrimoxazole for prophylaxis at the at health facility?’ Two categories were constructed yielding: (1) Yes, for respondents who reported having received cotrimoxazole and (2) No, for respondents who reportedly never received cotrimoxazole.

### Individual factors and health facility factors

The client’s age was assessed as a continuous variable and five categories were constructed for analysis of this variable. The client’s sex, residence, marital status and education level were assessed as binary variables. Three categories were constructed for religion and four categories for occupation. Distance (km) to the referral site was assessed as a continuous variable and three categories were constructed including: (1) 1 km – ≤2 km, (2) >2 km – ≤5 km, and (3) > 5 km.

### Community related factors

The community related factors were assessed as categorical variables. We used two closed ended questions: (1) did you receive any support from your family members (provision of transport to health facility, money to buy drugs or for laboratory tests, provision of food, escort client to health facility or any other related support) to help you access HIV care services?’ and (2) did you receive any support from the community members (sensitization on importance of seeking care, exemption from work to enable client seek care, provision of transport to health facility, adjusting work schedule to enable client seek care at the required time, any other assistance rendered to client to seek health care) to help you access HIV care at the referral facility?’. Two categorical variables were constructed for each one of the two questions 1 and 2 above yielding answers: Yes or No.

### Data management and analysis

The data was double entered into prepared data bases using MS Office Excel 2007 and univariable, bivariable and multivariable analysis done using STATA/SE 10.0 for windows software. Descriptive statistics was used and association between variables was determined using crude and adjusted odds ratios (OR) with 95 % confidence intervals (CI). For bivariable analysis, each independent variable was run against the outcome variable (reported receipt of cotrimoxazole) and associations with a p-value <0.05 were considered to be statistically significant. Multivariable analysis was done to control for confounding. Variables found to be statistically significant at bivariable level and the insignificant variables which were deemed to be important in influencing the outcome variable, were entered into the logistic regression model. Confounding was checked by observing whether variables included in the model caused a change in the odds ratio of the main exposure by at least 10 % and also by assessing whether variables that were insignificant at bivariable stage became significant at the multivariable level. A logistic model was constructed to determine the best model for prediction of access to HIV care services among the HIV positive clients. The best model was generated using forward stepwise multiple regressions and the fitness of the model assessed at every step using a log likelihood tending toward zero. The statistically significant adjusted OR’s were identified from the model and reported as independent factors associated with access to HIV care.

### Ethics statement

The study was approved by Makerere University School of Public Health, Higher Degrees Research and Ethics Committee and the Uganda National Council for Science and Technology. Permission to conduct the study in the area was obtained from the local administration of Kumi district. All participants provided written informed consent. Strict confidentiality was maintained during the conduct of the study. Access to the data was restricted to the investigators.

## Results

Out of a sample size of 352 a total of 350 respondents (141 males and 209 females) were traced, interviewed and included in the analysis. Eight sampled clients out of 352 were not found in their expected households; among whom, six had transferred. We successfully traced the six using information from their neighbors’ and through telephone calls. Two of the eight clients could not be traced at all.

### Socio-demographic characteristics of respondents

Most [59.7 % (209/350)] respondents were female, the mean age was 37.0 years, standard deviation of 8.6. Most [40.3 % (141/350)] of the respondents were in the age group 35–44 years, were married [59.7 % (209/350)], resided in rural areas [70.6 % (247/350)], and were peasant farmers [79.7 % (279/350)] (Table 1).

**Table 1:** Association between socio demographic, health facility, community related factors and access to HIV care services

Factors	Sub-category	Accessed HIV care (*n* = 284) No. (%)	Crude Odds Ratio (COR) (95 % CI)	Adjusted Odds Ratio (AOR) (95 % CI)
Social demographic				
Age group:	18–24	19 (73.1)	1	1
	25–34	103 (90.4)	3.4 (1.2–10.0)*	5.1 (1.5–17.1)**
	35–44	110 (78.0)	1.3 (0.5–3.4)	1.6 (0.5–4.7)
	45–54	42 (75.0)	1.1 (0.4–3.2)	1.4 (0.4–4.6)
	55–64	10 (76.9)	1.2 (0.2–5.8)	1.6 (0.3–8.9)
Sex:	Female	161 (77.0)	1	
	Male	123 (87.2)	2.0 (1.1–3.7)*	2.3 (1.2–4.4)*
Residence:	Rural	191 (77.3)	1	
	Urban	93 (90.3)	2.7 (1.3–5.6)**	2.5 (1.1–5.9)*
Education:	Never been to school	46 (79.3)	1	
	Ever school	238 (81.5)	1.1 (0.5–2.4)	
Marital status:	Not married	110 (78.0)	1	
	Married	174 (83.3)	1.4 (0.8–2.4)	
Occupation:	Peasant	223 (79.9)	1	
	Civil servant	12 (85.7)	1.5 (0.3–14.2)	
	Business	30 (90.9)	2.5 (0.7–13.3)	
	Others^1^	19 (79.2)	0.9 (0.3–3.4)	
Perceived benefit of HIV/AIDS information	No	217 (78.9)	1	
	Yes	67 (89.3)	2.2 (1.0–4.9)*	
Health facility				
Distance to health facility (km):	1 km – ≤2 km	187 (86.6)	1	1
	>2 km – ≤5 km	85 (71.4)	0.4 (0.2–0.7)*	0.6 (0.3–1.1)
	>5 km	12 (80.0)	0.6 (0.2–2.3)	1.2 (0.3–5.3)
Community				
Receipt of family support:				
	Yes	227 (84.4)	1	
	No	49 (71.0)	0.4 (0.2–0.8)*	0.5 (0.2–0.9)*
Receipt of community support:	Yes	210 (83.0)	1	
	No	66 (77.6)	0.7 (0.4–1.3)	

*p < 0.05, **p < 0.01, *CI* confidence interval, ^1^ skilled manual, unskilled manual and students

### HIV care services accessed at the health facilities

Out of the 350 respondents interviewed, [81.1 % (284/350) (95 % C.I: 76.0 %–85.1 %)] had reportedly received cotrimoxazole prophylaxis at the health centres, [61.1 % (214/350) (55.8 %–66.3 %)] received the Basic Care Package, [19.4 % (68/350) (95 % C.I: 15.4 %–24.0 %)] received ART, [37.4 % (131/350) (95 % C.I: 32.3 %–42.7 %)] accessed CD4 cell count testing services, [25.7 % (90/350) (95 % C.I: 21.2  %–30.6 %)] were screened for TB and [2 % (7/350) (95 % C.I: 0.8 %–4.0 %)] benefited from other laboratory services like malaria tests.

### Socio-demographic factors and access to HIV care services

At bivariable analysis, the age group of the respondents was found to be associated with the likelihood of accessing HIV care services. Respondents in the age group 25–34 years were 3.4 times more likely to access HIV care services than those in the age group 18–24 years and this association was statistically significant (COR = 3.4, 95 % CI, 1.2–10.0). Sex (males) and residence (urban) of the respondents were significantly associated with access to HIV care services (COR = 2.0, 95 % CI, 1.1–3.7 and COR = 2.7, 95 % CI, 1.3–5.6) respectively (Table 1).

### Health facility factors and access to HIV care services

At bivariable analysis, clients who resided at a distance of >2 km – ≤5 km from the health facility were less likely to access HIV care services than those who resided within a distance of two kilometers from the nearest health facility, and the findings were statistically significant (COR = 0.4, 95 % CI, 0.2–0.7).

### Relation between community factors and access to HIV care services

Majority [82.2 % (227/276)] of the respondents who accessed HIV care services received any form of support from their families. More than 60 % of the respondents who accessed HIV care services [62.3 % (172/276)] received food support, [54.7 % (151/276)] were escorted to the health facilities, while [41.0 % (113/276)] were helped with transport to the health facilities by their families. About [9.8 % (27/276)] received other forms of support like washing their clothes.

Respondents who did not receive support from their families were less likely to access HIV care services than those who received support from their families (COR = 0.4, 95 % CI, 0.2–0.8) (Table 1).

Seventy six percent [76.1 % (210/276)] of the respondents who accessed HIV care services received support from the community. Most [70.3 % (194/276)] of the respondents who accessed HIV care services reported that community sensitization on HIV/AIDS was the kind of support they received from their communities. The other forms of support received from the community were; adjusting of the clients’ work schedules to enable the clients access HIV care services [45.3 % (125/276)], provision with transport to the health facility [11.0 % (30.5/276)], counseling of client by friends [9.0 % (25/276)], exemption of client from duty to enable them access HIV care services [7.2 % (20/276)].

### Best fitting model for factors influencing access to HIV care services

After adjusting for confounding, the variables that remained significantly associated with access to HIV care services in a regression model included respondent’s age group 25–34 years compared to 18–24 years (AOR = 5.1, 95 % CI: 1.5–17.1); being male, (AOR = 2.3, 95 % CI: 1.2–4.4); urban residence (AOR = 2.5, 95 % CI: 1.1–5.9) and not receiving support from the family (AOR = 0.5, 95 % CI: 0.2–0.9). Distance of (>2 km – ≤5 km) to health facility (AOR = 0.5, 95 % CI: 0.3–1.1) and perceived benefit of obtaining information from health facility (AOR = 0.4, 95 % CI: 0.2–1.2) were not associated with uptake of HIV care services.

## Discussion

The majority of the participants we interviewed (81.1 %) received cotrimoxazole prophylaxis (access to HIV care). Access to HIV care in this population was considerably high. This could be attributed to the design and implementation of the HBCT program which ensured that HCT services were provided at people’s homes and HIV positive clients were referred to health facilities for care and treatment. The field teams, the peer educators through the drama groups and the CORPs encouraged clients to seek care and treatment at referral health facilities. Also the fact that the HBCT field teams ensured that they delivered the CD4 test results to client’s homes and subsequently encouraged the clients to seek care and treatment could have contributed significantly to the high access to HIV care services observed in this study.

Clients in the age group 25–34 years were more likely to access HIV care services compared to those in the age group 18–24 years. Literature on access to HIV care services disaggregated by age group is limited. However, a plausible explanation for this finding may be that individuals in this age group (25–34 years) are more familiar with the health care system compared with those in the age group (18–24 years) and so are able to navigate through the care system better.

The fact that males had better access to HIV care services compared to females was a surprising finding since other studies in Uganda [34, 35] and elsewhere [18, 36] indicates to the contrary. The reasons for this finding in this community could not be easily ascertained by this study. However, researchers have indicated that due to social norms on what is considered acceptable sexual behavior in women, women may face more stigma and discrimination than men [37, 38]. This may prevent some women from going to health facilities to access HIV care services. There is also a likelihood that women have to seek permission from their male partners [37], which is usually not the case with men since they are considered the decision makers in most relationships. This may also negatively affect the HIV care access patterns of women. One possible option to address this discrepancy is by encouraging the district health team and other district authorities to formulate and implement policies which affirmatively target females in this community, so as to ensure equitable access to HIV care services for both males and females.

Another observation was that urban residents were more likely to access HIV care services compared to rural residents. Researchers in Zimbabwe also found similar rural–urban disparities in access to HIV care services [39]. One possible explanation for our finding could be because both the private and public health care facilities in urban settings are physically easier to access. Tawiah, 2013 in a study of maternal health care in five sub-Saharan African countries found that rural women performed poorer than urban women in maternal health indicators and attributed this to physical inaccessibility of health services in most rural areas in African countries [40]. Related to physical access is distance to health facilities. Some studies have noted that distance to a health facility is an important factor in access to health services [21, 22]. The majority of respondents in our study resided within 5 km radius of a health facility. This implies that distance to facilities alone could not entirely explain the urban–rural disparity. It is also possible that other factors such as level of health facility, staffing norms, presence of ART clinics may have had an influence on the rural/urban disparity. However, we did not explore these factors in our study.

This study revealed that clients with a supportive family had better access to HIV care services than those without. The nature of family support that clients obtained was either food or being escorted to the health facility to access health care. In line with our findings, some researchers in South Africa also reported better uptake of HIV care and treatment among children who received family support. [41] However, Msellati et al. (2003) argued that the family may present a barrier to seeking care especially in instances where one needs to disclose their sero status to family members including their partner [25]. Our study did not ascertain the number of respondents who disclosed to their spouses. However, in a related study conducted in Kumi district by Kyaddondo et al. (2012), in which the majority of respondents were HIV negative, the researchers noted that all the HIV positive respondents had disclosed their test results to someone [32]. It is worth noting that by program design, the Kumi HBCT staff counseled and sensitized clients about the need for disclosure amongst other issues. This could have possibly impacted on the disclosure in the study population. Given our findings, we believe that community leaders and the district health team have a role to play in promoting family support for HIV positive clients. The opportunities for stakeholders to foster family support for HIV positive clients may be through holding village meetings, workshops and local radio talk shows. Another useful avenue may be through the use of family oriented organizations such as churches, mosques and non-governmental organizations (NGO’s).

One of the strength of this study is that it was conducted in a rural setting. Rural communities may have challenges in access to HIV care compared to urban communities [39]. Our study attempted to understand some of these factors from a rural perspective. Thus by addressing this crucial information gap through this study, we hope that programs offering HBCT services in similar settings may make use of our findings to develop strategies for improving access to HIV care and treatment services for their clients. The other strength is that this was a population based study. Most studies in our setting have been conducted at health facilities. This study provides an opportunity to understand HIV care and treatment services access factors from a population level perspective.

The study had some limitations. It was conducted in January and February 2009, almost nine months after the Kumi HBCT program closure in March 2008. There was thus a likelihood of recall bias from the respondent’s responses. We tried to minimize this bias by using skilled and experienced interviewers and ensuring additional probing to elicit the necessary information. Researchers agree that the skills of the interviewer and the way questions are asked play a key role in obtaining accurate responses.

Our main outcome variable was measured by reported receipt of cotrimoxazole for prophylaxis. Data obtained through self-reports method is prone to recall and social desirability bias. We were unable to validate findings from our data with official clinic records at the health facilities because this was a population based survey. However, self- reports have been used before by other researchers [42]. We categorized ‘education’ into only two sub-categories of ‘ever been to school’ and ‘never been to school’. This categorization was too broad and may have masked the effect of education if any by combining individuals with several years of schooling with those with few years of schooling.

## Conclusions

There was relatively high access to HIV care and treatment services at health facilities for the HIV positive clients who were referred from the Kumi home based HIV counseling and testing program. The factors that were associated with access to HIV care services include; age group, sex, residence and having a supportive family. Stakeholders involved in providing HIV care and treatment services in similar settings should therefore consider these socio-demographic variables as they formulate interventions to improve access to HIV care services in their settings.

## Additional file

Additional file 1:
**HIV positive clients’ questionnaire.** (DOCX 19 kb)
