# Supplementary material for: Factors associated with access to HIV care services in eastern Uganda: the Kumi home based HIV counseling and testing program experience
Source: BMC Fam Pract. 2015 Nov 3;16:162. doi: 10.1186/s12875-015-0379-6 (PMC4630893; doi:10.1186/s12875-015-0379-6)
Supplement: Additional file 1: — HIV positive clients’ questionnaire. (DOCX 19 kb) [file 12875_2015_379_MOESM1_ESM.docx]

**APPENDIX I: HIV POSITIVE CLIENTS’ QUESTIONNAIRE**

| **IDENTIFICATION** | |
| --- | --- |
| Questionnaire number |  |
| Date of interview |  |
| County |  |
| Sub County |  |
| Parish |  |
| Village |  |
| **INSTRUCTIONS: FILL IN THE SPACES OR CIRCLE THE CHOICE AS APPROPRIATE** | |
| **Individual factors** | |
| 1. What is the age of the client referred in completed years | ……………………… |
| 2. What is the sex of the client referred? | 1) Male  2) Female |
| 3. What is the marital status of the client referred | 1) Married  2) Single  3) Separated  4) Co-habiting  5) Widowed  6) Divorced |
| 4. What is the religion of the client | 1) Anglican  2) Moslem  3) Born again  4) Traditional  5) Roman Catholic  6) SDA  7) Others,specify………… |
| 5. Where do you reside? (Interviewer has to confirm using predetermined list of rural and urban centres) | 1) Rural (Village)  2) Urban (Town) |
| 6. What is the highest level of education attained by the client referred? | 1) None  2) Lower Primary (P1-4)  3) Upper Primary (P5-7)  4) Secondary (S 1-4) 5) Higher school (S 5-6)  6) Post -secondary institution or university |
| 7. What is the occupation of the client referred? | 1) Peasant farmer  2) Civil servant  3) Skilled manual  4) Unskilled manual  5) Commercial farmer  6) Business person 7) other, please specify………………. |
| 8. Do you know of any signs and symptoms of AIDS disease in people infected with HIV? | 1) Yes 2) No |
| 9. Do all people infected with HIV virus have signs and symptoms? | 1) Yes 2) No |
| 10. Is it always important to seek medical care even when you do not have signs and symptoms of HIV? | 1) Yes 2) No |
| 11. Are there any benefits of seeking HIV medical care once you have been referred by a health worker, even when you do not feel ill at that moment in time? | 1) Yes (go to qn.12, then continue with 13)  2) No (go to qn.13) |
| 12. Can you mention any benefits associated with seeking HIV medical care, even when you do not feel ill at that moment in time?( Interviewer circles answer given appropriately) | 1) Avoidance of diseases associated with HIV  2) Prolonging life  3) Improving the quality of life  4) Avoid medical expenses incurred in treatment of diseases associated with HIV  5) Obtaining information on HIV/AIDs  6) Other reason, please specify …………………….. |
| **Health facility factors** | |
| 13. Were you referred to a health facility for HIV care? | 1) Yes (go to qn 14) 2) No |
| 14. Why were you referred (circle as appropriate)? | 1) For laboratory services (CD4, TB screening)  2) For drugs (cotrimoxazole, ART, PMTCT)  3) For both laboratory and drugs |
| 15. Did you receive cotrimoxazole at the health facility that you were referred to? | 1) Yes  2) No |
| 16. Did you receive laboratory services at the health facility that you were referred to? | 1) Yes  2) No |
| 17. Did you go to the health facility soon after being referred? | 1) Yes (go to question 19) 2) No (go to question 18) |
| 18. Why didn’t you go to the health facility soon after being referred? | 1) Lack of transport 2) Long distance to health facility  3) Lack of knowledge on the importance of visiting the health facility  4) Lack of or limited services at the health facility  5) Household demands  6) Work or job demands 7) Social stigma  8) High cost of services at the health facility 9) Lack of privacy at the health facility  10) Rudeness of the health workers at the health facility  11) Other reason (specify)…………………… |
| 19. After how long did you visit the health facility? | 1) On the date of referral  2) Within 1 week of referral 3) within 1 month of referral  4) More than 1 month after referral |
| 20. Were the laboratory services for which you were referred available at the at referral site? | 1) Yes 2) No |
| 21. Were the drugs for which you were referred available at the at referral site? | 1) Yes 2) No |
| 22. What specific services did you receive at the referral health facility? | 1) HIV testing 2) Basic care package  3) TB screening 4) ART  5) CD4 count testing 6) Cotrimoxazole prophylaxis  7) PMTCT 8) Other, please specify……… |
| 23. Did you find the health workers at referral site helpful? | 1) Yes 2) No |
| 24. Do you think services at referral site were affordable? | 1) Yes 2) No |
| 25. What is the approximate distance in kilometers from your home to the facility to which you were referred to? | ……………………..Km |
| 26. What means of transport did you use to get to the referral facility? | 1) Walked to the facility  2) Used a Bicycle  3) Used Public means (taxi)  4) Used a personal vehicle 5) Used a Motorcycle  6) Other, please specify…………… |
| 27. How much do you estimate the cost of the journey to and fro the referral facility? | 1)………………………………………Uganda shillings |
| 28. How did the health workers treat you when you visited the referral facility? | 1) Poorly 2) Well  3) Very well |
| 29. How do you rate the conduct of the health workers at the referral facility? | 1) Not supportive 2) Supportive  3) Very supportive |
| **Community related factors** | |
| 30. Did you receive any support from your family members to help you access HIV care services? | 1) Yes (go to qn31) 2) No (go to qn32) |
| 31. What kind of support did you receive from your family members in order to access HIV care services? | 1) Provided transport to health facility 2) Provided money for buying drugs  3) Provided money for laboratory tests 4) Escorted me to health facility  5) Provided food while at the health facility 6) Others, specify………………… |
| 32. Did you receive any support from the community members to help you access HIV care services at the referral facility | 1) Yes (go to qn 33) 2) No (End of interview. Thanks for your time) |
| 33. What kind of support did you receive from the community in order to access HIV care services? | 1) Provided community sensitization on importance of seeking HIV care  2) Exempted me from work to help me get time to seek HIV care  3) Provided transport means to the health facility  4) Adjusted my work schedule to enable me seek medical care at the required time  5) Others, specify…………………………………………………………… |
| **END OF INTERVIEW , THANKS A LOT FOR YOUR TIME** | |
